# Whole-genome sequencing of bloodstream *Staphylococcus aureus* isolates does not distinguish bacteraemia from endocarditis

**DOI:** 10.1099/mgen.0.000138

**Published:** 2017-11-20

**Authors:** Berit Lilje, Rasmus Vedby Rasmussen, Anders Dahl, Marc Stegger, Robert Leo Skov, Vance G. Fowler, Kim Lee Ng, Kristoffer Kiil, Anders Rhod Larsen, Andreas Petersen, Helle Krogh Johansen, Henrik Carl Schønheyder, Magnus Arpi, Flemming S. Rosenvinge, Eva Korup, Ulla Høst, Christian Hassager, Sabine Ute Alice Gill, Thomas Fritz Hansen, Thor Bech Johannesen, Jesper Smit, Peter Søgaard, Paal Skytt Andersen, Niels Eske-Bruun

**Affiliations:** ^1^​ Department of Bacteria, Parasites and Fungi, Statens Serum Institut, Copenhagen, Denmark; ^2^​ Department of Cardiology, Copenhagen University Hospital, Herlev-Gentofte, Copenhagen, Denmark; ^3^​ Division of Infectious Diseases, Duke University Medical Center, Durham, NC, USA; ^4^​ Department of Clinical Microbiology, Copenhagen University Hospital, Rigshospitalet, Copenhagen, Denmark; ^5^​ Department of Clinical Microbiology, Aalborg University Hospital, Clinical Institute, Aalborg University, Aalborg, Denmark; ^6^​ Department of Clinical Microbiology, Copenhagen University Hospital, Herlev-Gentofte, Herlev, Denmark; ^7^​ Department of Clinical Microbiology, Odense University Hospital, Odense, Denmark; ^8^​ Department of Cardiology, Aalborg University Hospital, Aalborg, Denmark; ^9^​ Department of Cardiology, Copenhagen University Hospital, Rigshospitalet, Copenhagen, Denmark; ^10^​ Department of Cardiology, Odense University Hospital, Odense, Denmark; ^11^​ Department of Clinical Microbiology, Aalborg University Hospital, Aalborg, Denmark; ^12^​ Department of Cardiology, Clinical Institute, Aalborg University, Aalborg University Hospital, Aalborg, Denmark; ^13^​ Department of Animal and Veterinary Sciences, University of Copenhagen, Copenhagen, Denmark; ^14^​ Translational Genomics North, Flagstaff, USA; ^15^​ Department of Cardiology, Copenhagen University Hospital, Herlev-Gentofte, Clinical Institute, Aalborg University, Copenhagen, Aalborg, Denmark

**Keywords:** genome-wide association, *Staphylococcus aureus*, bacteraemia, infective endocarditis

## Abstract

Most *Staphylococcus aureus* isolates can cause invasive disease given the right circumstances, but it is unknown if some isolates are more likely to cause severe infections than others. *S. aureus* bloodstream isolates from 120 patients with definite infective endocarditis and 121 with *S. aureus* bacteraemia without infective endocarditis underwent whole-genome sequencing. Genome-wide association analysis was performed using a variety of bioinformatics approaches including SNP analysis, accessory genome analysis and k-mer based analysis. Core and accessory genome analyses found no association with either of the two clinical groups. In this study, the genome sequences of *S. aureus* bloodstream isolates did not discriminate between bacteraemia and infective endocarditis. Based on our study and the current literature, it is not convincing that a specific *S. aureus* genotype is clearly associated to infective endocarditis in patients with *S. aureus* bacteraemia.

## Abbreviations

CC, clonal complex; IE, infective endocarditis; MLST, multilocus sequence typing; MRSA, methicillin resistant Staphylococcus aureus; MSSA, methicillin sensitive Staphylococcus aureus; PCA, principal component analysis; SAB, Staphylococcus aureus bacteraemia; SNP, single-nucleotide polymorphism; SRA, Sequence Read Archive; ST, sequence type; WGS, whole-genome sequencing.

## Data Summary

1. Read data is deposited in the European Nucleotide Archive (ENA): study accession number ERP023934 (http://www.ebi.ac.uk/ena/data/view/ERP023934).

2. Code, R-scripts and data files used when running the scripts can be accessed at Sourceforge, https://sourceforge.net/projects/sabvsie/files/.

## Impact Statement

We have analysed the *Staphylococcus aureus* genomes in isolates from 121 cases of bacteraemia and 120 cases of adult infective endocarditis patients. The aim has been to investigate possible genetic associations to either condition. Previous studies in this field have reported conflicting results due to limitations of methods available at the time. By applying extensive bioinformatics analyses on whole-genome sequenced data, we were able to explore genetic associations with a much less biased approach.

Overall, our findings indicate that there is no obvious genetic difference between bacteraemia and infective endocarditis isolates. Future studies using large collections isolates from selected lineages may identify weaker associations.

## Introduction

Infective endocarditis (IE) is the most feared complication of invasive staphylococcal infections occurring in 22–25 % of patients with *Staphylococcus aureus* bacteraemia (SAB) [1, 2]. During the past two decades, there has been an increasing effort to discover why some *S. aureus* isolates tend to cause more invasive infections than others. One of the key questions is, how strong is the link between bacterial genotype and clinical manifestations? So far, several different genetic methods have been applied with conflicting results. Some studies have investigated *S. aureus* isolates from nasal carriers compared to isolates from mixed invasive infections using polymerase chain reaction (PCR) [3–6]. The results from these studies range from no associations through subtle single gene differences to conflicting results on clonal complex (CC) association. Other studies have used PCR and DNA microarrays to compare isolates from skin and soft tissue infections (SSTI) with isolates from infective endocarditis (IE), identifying associations between CCs, between different genes and IE [7, 8]. Furthermore, studies comparing SAB isolates to IE isolates in methicillin-resistant *S. aureus* (MRSA) [9], methicillin-sensitive *S. aureus* (MSSA) [10] and community-acquired (CA) bacteraemia [11] have reported conflicting results on associations to IE. Common for all the above studies is that they have searched for absence vs presence in specific genetic areas in the *S. aureus* genome using PCR or microarray.

Genome-wide association studies (GWAS) have recently been applied to bacterial genome data and may be used to identify genes or gene variants that may be associated with a measured or observed phenotype. Several different approaches have been used to elucidate association ranging from single nucleotide polymorphisms [12, 13] to k-mer based studies [14–17]. So far, these have been focusing on a very well-defined phenotype, either toxicity as a proxy for virulence [12, 13], host specificity [14], or antibiotic resistance [15, 17, 18]. All studies acknowledge the challenge concerning clonality of different species that may mask potential association either by only investigating single clones independently [12–14] or by adjusting for population structure [16, 19]. In the clinical laboratory with a plethora of clonal lineages, it would be of great advantage if specific genes, gene variants or combination of genes could be used as predictors for secondary infections such as IE. Therefore, using GWAS tools on whole-genome sequencing (WGS) from *S. aureus* blood culture samples could potentially identify such predictors at an early stage and allow for more intensive treatment of patients with increased risk of developing IE.

No previous studies have examined differences in *S. aureus* isolates from patients with or without IE using WGS data. The present study used WGS and GWAS analysis to search for possible genetic differences in *S. aureus* isolates from patients with SAB who never had secondary complications with isolates from patients who developed IE subsequent to the initial bloodstream infection.

## Methods

### Patients

Two prospective studies were combined to identify patients for this study. From study I [20] we identified 84 patients with definite *S. aureus* IE fulfilling the modified Duke Criteria [21]. From study II [2] we identified additional 36 patients with definite *S. aureus* IE and 121 patients without endocarditis (SAB-only), all investigated by echocardiography. To reduce the risk of overlooking the IE diagnosis, the patients were followed up for at least 30 days after discharge. No patients were readmitted with suspected IE. No patients were intravenous drug users, and all suffered from MSSA infections. The patients from the two groups are referred to as IE and SAB-only patients, respectively.

Additionally, we performed a subgroup analysis on isolates from CA bacteraemia [22] excluding prosthetic valve IE in order to reduce confounding risk factors. This sub-analysis included 67 patients with CA native valve IE and 40 patients with CA SAB-only out of the 241 patients.

### DNA sequencing and *S. aureus* typing

Isolates from all patients were received at the time of diagnosis and immediately stored at −80 °C. DNA was purified using Qiagen DNeasy Blood and Tissue Purification Kit (Qiagen).

Whole-genome sequencing was performed using paired-end (2×250 bp) sequencing on a MiSeq instrument (Illumina) to an average sequencing depth of at least 50. Assemblies were performed using a pipeline based on SPAdes v3.5.0 [23] with settings for Illumina 250 bp paired-end reads. Sequence types (ST) were determined using the program MLST v1.2 (https://github.com/tseemann/mlst) with subsequent CC grouping using eBURST (www.mlst.net).

### Bioinformatics analysis

#### Single nucleotide polymorphisms (SNPs)

Briefly, core SNPs were identified by aligning the sequence data from each isolate against the chromosome of the CA-347 ST45 reference chromosome (GenBank accession no. CP006044) [24] using the short-read alignment component of the Burrows-Wheeler Aligner (BWA) [25] after the removal of duplicated regions in the reference using NUCmer [26, 27]. Each alignment was analysed for SNPs using NASP [28]. All SNPs that did not meet a minimum coverage of 10 or if the variant was present in <90 % of the base calls were excluded. For downstream analysis, all bases identical to the reference were set to ‘0’, while all bases differing from the reference base were set to ‘1’. (In approximately 3 % of identified SNP positions more than two different bases were observed; here, all sequences different from the reference were considered as ‘1’). Phylogeny on the identified SNPs was inferred using the maximum-likelihood approximation in FastTree v2.1.5 [29] as implemented in Geneious v9.1.3 [30] (Biomatters) and visualized using iTool v3.2.4 [31, 32].

#### SNP analysis

Univariate analysis was performed using Fisher’s exact test. Multivariate comparison was performed using ‘*Differential Analysis of Principal Components*’ (DAPC) [33] from the R package ‘*adegenet*’ [34]. To find the optimal number of principal components (PCs) to use for DAPC, multiple cross-validations were performed through the xvalDapc function. If no optimal number of PCs compared to random sampling was found, the analysis was terminated. Correction for population stratification was performed by regression along the CCs for each SNP and keeping the residuals for further analysis. After correction for population stratification, a Mann-Whitney U test was used for univariate analysis.

#### Accumulation of SNPs

Accumulations of SNPs in genes were compared using the CA-347 reference chromosome with the program ‘*Tool for Rapid Annotation of Microbial SNPs*’ (TRAMS) v1.0.2 [35] with default settings. Furthermore, to include non-coding regions, SNP positions were scanned using a sliding window of 1000 bp with increments of 1 bp. For each window position the number of SNPs per isolate was determined. The number and distribution of SNPs were compared between the two groups. In order to limit the number of statistical tests, only windows where the log_2_FC (log_2_ of fold changes) between the mean number of SNPs in the two groups were >0.5 or <−0.5 were assessed.

#### Accessory genome

Prokka v1.2 [36] was used to identify and annotate open reading frames in the assembled genomes with defaults settings. Roary v3.6.0 [37, 38] was applied to define gene ‘presence/absence’ across the isolate collection. Uni- and multivariate analyses were performed as described above. Selected sequences were extracted to confirm results using Megablast and tblastx as implemented in Geneious v9.0.4 (Biomatters). Additionally, known virulence factors where identified using VirulenceFinder [39] and investigated for correlation with IE.

#### K-mer analysis

All assembled contigs were broken down to a fragment (k-mer) size of 30 bps and added to a dictionary in python v2.7.10 similar to the methods applied by Earle and co-workers [15]. The number of occurrences of each unique k-mer (or reverse complement of the k-mer) was noted. Each k-mer was only counted once per sample to avoid samples with long k-mer repeats skewing the results. K-mers did not span contig junctions. The presence of each k-mer in SAB-only and IE samples was compared.

#### Within-group variation and convergent evolution

The CCs most frequently found in the data set, CC45 (*n*=52), CC30 (*n*=45), CC15 (*n*=31), CC1 (*n*=19), CC5 (*n*=17), and CC8 (*n*=14), were tested individually for associations within the group between phenotype and both SNPs and presence/absence of accessory genes. The possibility of convergent evolution giving rise to genotypes associated with IE was specifically addressed by testing only SNPs where both SNP variants were found in at least two of the six main CCs as well as genes where both presence and absence was observed within at least two of the CCs.

### Statistics

R software (v3.2.4) was used for all statistical analyses. Unless otherwise stated, proportion tests were performed by Fisher’s exact test, while comparisons of distributions were tested using Mann-Whitney U test. *P*-values were corrected for multiple testing using the false discovery rate (FDR) and Bonferroni correction methods, and the significance level was set to 0.05. Unless otherwise stated, the R package ‘ggplot2’ v2.1.0 [40] was used for visualisations.

### Statistical power considerations

To ensure that sufficient statistical power was obtainable in our data set with the high number of SNPs identified (120 850 SNPs), all combinations of SNPs (ranging from a SNP being present in (i) all IE samples and no SAB-only samples to (ii) no IE samples and all SAB-only samples) were calculated and corrected for multiple testing. Fig. S1 (available in the online Supplementary Material) gives an overview of SNP patterns that would yield significant *P*-values, after multiple testing corrections using Bonferroni. Based on this simulation, a SNP present in 90 of 120 (75 %) IE isolates and 50 of 121 (41 %) SAB-only isolates would show a significant difference (proportion difference 34 %, *P*<0.05). Another example of significant combination would be a SNP present in 62 of 120 (52 %) IE isolates and 100 of 121 (83 %) SAB-only isolates (proportion difference 31 %, *P*<0.05). As the power plot illustrates, any combination with larger difference between IE isolates and SAB-only isolates would result in *P*-values below 0.05 after Bonferroni correction. Consequently, the study is powered to detect a SNP proportion difference around 30 % comparing IE isolates to SAB-only isolates.

**Fig. 1.**
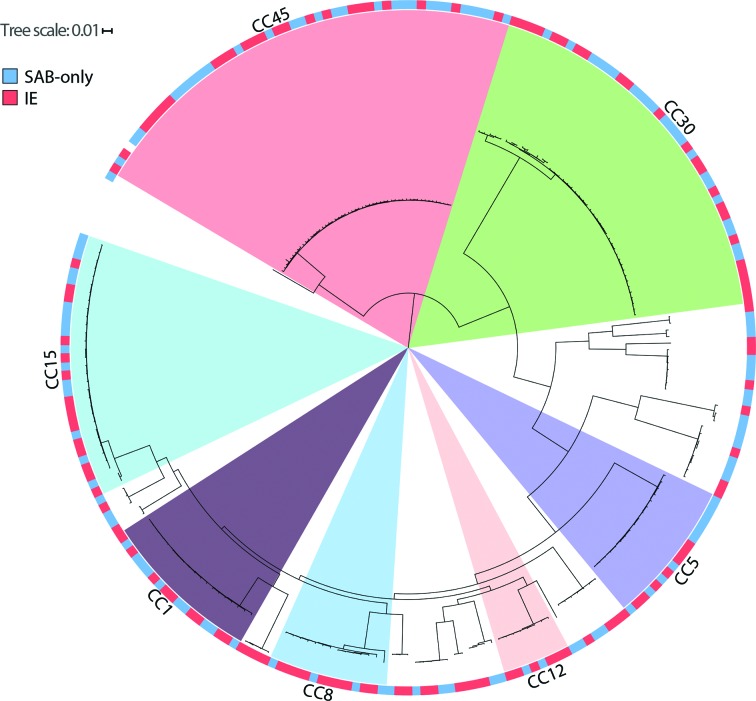
Rooted phylogenetic analysis using a maximum likelihood approximation on the 120 850 SNPs identified in the conserved core genome of the 241 *S. aureus* isolates. The tree is rooted on the CC45 branch [49] and CC groups with more than 10 isolates are highlighted. Outer circle corresponds to infection type (red=IE, blue=SAB-only). For identification of specific samples see Fig. S5.

## Results

One hundred and twenty IE patients and 121 SAB-only patients were investigated. There was an insignificant trend towards IE patients being younger and more often male than SAB-only patients. One *S. aureus* isolate from each patient was investigated by whole-genome sequencing.

### Bacterial genotyping

Twenty-two CCs were represented among the 241 *S. aureus* isolates. The major CCs were: CC45, CC30, CC15, CC1, CC5 and CC8, which combined accounted for 74 % of the samples. No single CC was significantly associated to SAB-only or to IE, neither in the entire cohort nor in the subgroup of CA infections (Table 1).

**Table 1.** Prevalence of clonal lineages among SAB-only and IE patients including subset of patients with CA infection

	**All patients**			**Patients with community acquired (CA) infection**		
**CC type**	**SAB-only (120)**	**IE (121)**	***P* value**	**CA SAB-only (40)**	**CA IE (67)**	***P* value**
CC45	28 (23 %)	24 (20 %)	0.64	5 (13 %)	16 (24 %)	0.21
CC30	22 (18 %)	23 (19 %)	0.87	9 (23 %)	11 (16)	0.45
CC15	16 (13 %)	15 (13 %)	1.00	4 (10 %)	11 (16 %)	0.40
CC1	10 (8.3 %)	9 (7.5 %)	1.00	3 (7.5 %)	3 (4.5 %)	0.67
CC5	10 (8.3 %)	7 (5.8 %)	0.62	7 (18 %)	4 (6.0 %)	0.10
CC8	4 (3.3 %)	10 (8.3 %)	0.11	2 (5 %)	3 (4.5 %)	1.00
CC12	2 (1.7 %)	6 (5.0 %)	0.17	0 (0 %)	5 (7.5 %)	0.15
CC22	5 (4.1 %)	3 (2.5 %)	0.72	3 (7.5 %)	2 (3.0 %)	0.36
CC25	3 (2.5 %)	4 (3.3 %)	0.72	2 (5.0 %)	2 (3.0 %)	0.63
CC59	5 (4.1 %)	2 (1.7 %)	0.45	1 (2.5 %)	1 (1.5 %)	1.00
CC7	3 (2.5 %)	2 (1.7 %)	1.00	1 (2.5 %)	0 (0 %)	0.37
CC188	0 (0 %)	4 (3.3 %)	0.06	0 (0 %)	3 (4.5 %)	0.29
CC9	2 (1.7 %)	2 (1.7 %)	1.00	0 (0 %)	1 (1.5 %)	1.00
CC20	2 (1.7 %)	1 (0.8 %)	1.00	1 (2.5)	1 (1.5)	1.00
CC509	2 (1.7 %)	1 (0.8 %)	1.00	0 (0 %)	1 (1.5 %)	1.00
CC97	1 (0.8 %)	2 (1.7 %)	0.62	0 (0 %)	1 (1.5 %)	1.00
CC121	2 (1.7 %)	0 (0 %)	0.50	0 (0 %)	0 (0 %)	na
CC182	1 (0.8 %)	1 (0.8 %)	1.00	1 (2.5 %)	0 (0 %)	0.37
CC573	1 (0.8 %)	1 (0.8 %)	1.00	1 (2.5 %)	1 (1.5 %)	1.00
CC6	0 (0 %)	2 (1.7 %)	0.25	0 (0 %)	1 (1.5 %)	1.00
CC50	0 (0 %)	1 (0.8 %)	0.50	0 (0 %)	0 (0 %)	na
CC72	1 (0.8 %)	0 (0 %)	1.00	0 (0 %)	0 (0 %)	na
Singleton	1 (0.8 %)	0 (0 %)	1.00	0 (0 %)	0 (0 %)	na

### Genome-wide association analysis

A summary of the overall bioinformatics analyses applied in the study is shown in Fig. S2.

#### Investigation of association to single SNPs or combination of SNP

**Fig. 2.**
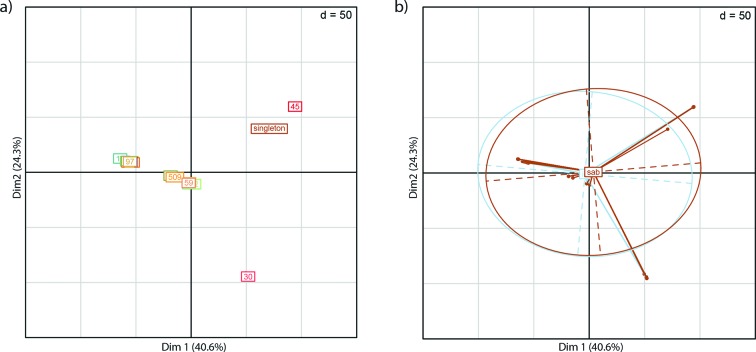
Principal Component Analysis (PCA) plots showing the relatedness of the 241 isolates. PCA plots were made with the dudi.pca function in R retaining 2 axes. (a) PCA of all 241 samples, showing the two first principal components, samples are coloured by CC type. (b) The same PCA plot as in (a), but coloured by infection type. Taken together this shows that the strongest signal in the data derives from the CCs with dense clusters and not the infection type. Associated eigenvalue plots are shown in Fig. S6.

**Fig. 3.**
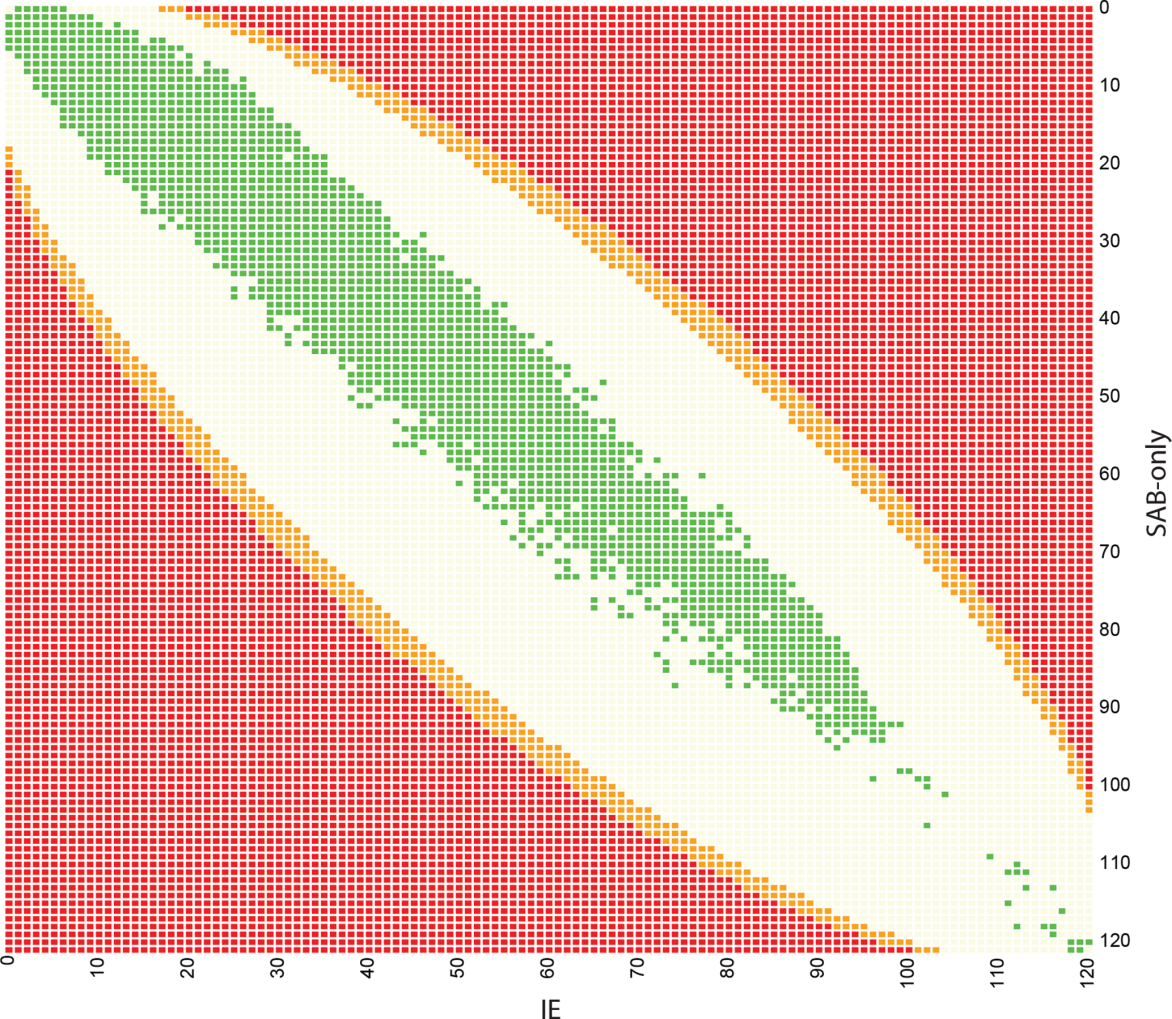
SNP distribution compared to significance level. The colour of the square corresponds to significance level (as in Fig. S1). Red squares correspond to a *P*-value <0.05 after multiple testing correction, orange squares correspond to *P*-values >0.05 and <1 after correction, light yellow squares correspond to *P*-values=1 after multiple testing correction. All 120 850 SNPs from our dataset are marked in green. This illustrates that no SNPs in this project are significantly associated to either IE or SAB-only isolates, based on Fisher tests, and a significance level of 0.05 after multiple testing correction.

To investigate whether single or combination of SNPs could discriminate between isolates from SAB-only and IE isolates, a total of 120 850 identified SNPs using NASP were examined. The relatedness of the isolates was inferred in a phylogeny with the IE association and most common CCs highlighted in Fig. 1 and as a principal component analysis (PCA) plot coloured by CC (Fig. 2a) and coloured by IE association (Fig. 2b). The association of each variable position to SAB-only or IE was investigated and no single SNP showed any statistical overrepresentation in either group (Fig. 3).

Multivariate analysis (DAPC) was performed to investigate if any combination of SNPs could predict IE vs SAB-only. The first step of DAPC analysis consists of finding the optimal number of PCs to retain. None of the tested PCs (Fig. S3a) could predict IE vs SAB-only better than random, which demonstrated a lack of signal in the data, and as a consequence the analysis was terminated.

As principal component analysis revealed that the strongest signal in the dataset corresponded to CCs of the isolates, correction for population stratification was performed (Fig. S3b), followed by univariate and multivariate analysis, which did not identify any significant hits (Fig. S3c). All the above analyses were also insignificant in the subgroup of CA infections.

#### Accumulation of SNPs in core genes or regions

Certain regions or genes may be prone to accumulation of variations [41], but if these were located at different positions in a given gene or region in different isolates such an accumulation would not be picked up by the methods applied above. TRAMS analysis was therefore performed on all annotated genes in the CA-347 reference, but no association to accumulation of SNPs was observed. Furthermore, in order to include non-coding and non-annotated regions of the core genome a 1000 bp sliding window was applied, but without identification of any accumulation of SNPs when comparing the two outcomes. Fig. S4 shows a Manhattan plot comparing the number of SNPs in 1000 bp bins between SAB and IE samples, the *P*-value is based on a two-sided *t*-test.

#### Mutations in non-core genome

To assess if variable positions outside the core genome contained associated SNPs, information on these were extracted for each position in the chromosome according to the reference, including uncalled bases. First, we investigated if any positions (including non-SNP positions) had more uncalled bases in SAB-only or IE isolates. This was not found to be the case. Secondly, for each position containing both SNPs and uncalled bases among all isolates, proportions of the SAB-only and IE samples belonging to either of the categories ‘SNP’, ‘non-SNP’ and ‘uncalled’ was investigated with no significant differences.

#### Gene presence/absence

**Fig. 4.**
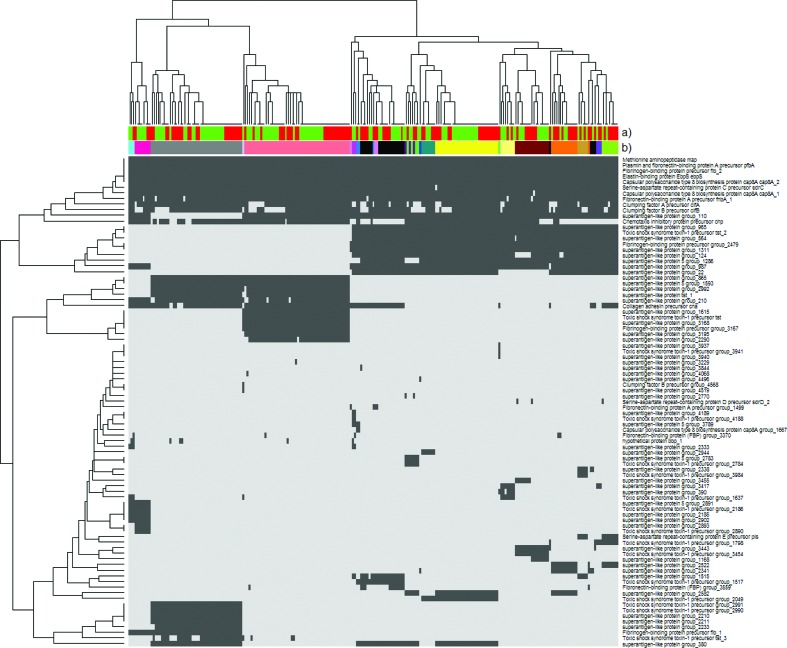
Presence (dark grey) and absence (light grey) of known virulence genes. (a) Infection type is indicated [IE (red) or SAB-only (green)]; (b) CC of the isolate. None of the virulence genes identified were found to be associated with IE or SAB-only.

Genetic content is often associated to virulence and as such we investigated the accessory genetic content using all identified genes in all included isolates using the Roary results. This showed that no genes were significantly associated to either IE or SAB-only. To see if a combination of genes could explain the outcome, DAPC analysis was performed. As with the SNP data, no positive signal was identified, and no further DAPC analysis was performed. Fig. 4 shows presence and absence of virulence genes identified by Prokka [36] and VirulenceFinder [39], and how they relate to CCs.

#### Within-group variation and convergent evolution

For within-group variation no single SNP or gene was found to be associated with IE when examining the six largest CCs individually. Convergent evolution was addressed by testing SNPs from sites where variants or genes were present or absent in at least two CCs. The number of SNPs tested reduced from 120 850 to 510 and the number of genes tested reduced from 3326 to 843, still no significant associations were found.

#### K-mer over-representation

Despite using a variety of tools to investigate associations to IE or SAB-only, they all depend on either a reference sequence to define SNPs or on the accuracy of gene identification. To assess if any differences without such assumptions exist, we investigated if any DNA sequence from the *de novo* assemblies was overrepresented in either outcome. All SAB-only and IE samples were broken down to k-mers of 30 bp; and each k-mer was investigated for over-representation in either group of isolates. No significant association was found to SAB-only or IE.

## Discussion

The current study investigated genetic differences in *S. aureus* isolates from IE patients compared with SAB-only patients using unbiased genome-wide association analyses that have been successfully applied in studies with well-defined bacterial phenotypes such as antibiotic resistance [19] or virulence [12]. We performed analyses of the entire cohort as well as a subgroup with native valve, community-acquired infections. We did not discover any significant associations between CC, individual SNPs, combination of SNPs, or absence/presence of genes and either SAB-only or IE.

The previous knowledge in this field is characterised by comparison of different clinical patient groups, varying genetic methods and conflicting results (Table 2). When analysing CCs, three studies found no association to IE [5, 10, 42], two studies found CC30 to be associated to increased invasiveness [4, 7], while another study [6] found the opposite with CC30 being associated to nasal carriage and not to invasive disease. Additionally, two studies have shown CC8 to be associated to intravenous drug abuse and right-sided IE [9, 10]. In other studies entirely different CCs were associated to invasiveness and IE, ranging from CC5 in MSSA [4], CC12 and CC20 in a small study with 24 IE patients [8], and CC15 in CA infections [11]. Investigations of associations between phenotype and specific virulence genes have also shown contradictory results as shown in Table 2. In line with our study, Tristan *et al*. reported no specific virulence factors or genotypes significantly associated with IE when comparing 81 MSSA SAB-only isolates to 89 MSSA IE isolates [10]. Bouchiat *et al*. investigated 54 CA SAB-only vs 72 CA native valve isolates and found no univariate genetic markers of IE, but a combination of eight specific genes was associated to IE [11]. A study by Young *et al.* applied WGS to identify changes in a MSSA isolate from a nasal swab in the transition to bloodstream infection in the same patient [43]. They identified numerous mutations with 30 SNPs and several deletions and insertions indicating that genetic changes may take place during the course of infection [43]. To our knowledge, the present study is the first to use WGS to compare larger groups of IE isolates with SAB-only isolates, where all patients have undergone echocardiography. Our comprehensive analysis of the bacterial genomes identifying no genetic association suggests that the major factor that determines the outcome of an *S. aureus* bloodstream infection is host susceptibility. Transition from carriage to bloodstream infection and further on to IE may thus rather be due to host genetics and host immune responses than caused by specific traits of the infecting bacterium. In support of this, we have recently reported a higher incidence of *S. aureus* bacteraemia among first degree relatives of *S. aureus* patients compared to the general population, suggesting that there is a host genetic component, partly explaining why some individuals are more prone to acquire *S. aureus* bacteraemia than others [44]. Specific host gene variants have also been associated to susceptibility to endocarditis [45–48].

**Table 2.** Summary of findings from other studies examining associations between SA virulence and specific genes, CCs

**References**	**Patients**	**Genetic analysis**	**Multiple testing correction**	**Findings**
Peacock *et al.* [3]	179 *S. aureus* nasal carriers vs 155 *S. aureus* invasive disease	PCR	None	Invasive *S. aureus* associated to 7 genes: *fnbA, cna, sdrE, sej, eta, hlg, icaA*.
Feil *et al.* [5]	179 *S. aureus* nasal carriers vs 61 invasive CA *S. aureus* infections vs 94 hospital acquired invasive *S. aureus* infections	MLST	None	No association between CC and invasiveness of infection.
Fowler *et al.* [4]	118 *S. aureus* nasal carriers vs 104 uncomplicated SAB and STI* vs 157 IE and ostitis	MLST+PFGE+PCR	None	CC5 associated to invasiveness in MSSA and CC30 associated to invasiveness in MRSA.
Lalani *et al.* [9]	65 MRSA SAB vs 24 MRSA IE	PFGE+PCR for 33 virulence genes	FDR (20 %)	USA300 (CC8) MRSA bacteraemia associated to intravenous drug use and right sided IE. Persistent MRSA SAB associated to *seg* gene. Non-persistent MRSA SAB associated to *pvl* gene.
Nienaber *et al.* [7]	114 *S. aureus* STI* vs 114 IE	MLST+PCR	FDR (10 %)	IE associated to CC30 and 5 genes: *cna, map/eap, tst, sei, sdrC*.
Tristan *et al.* [10]	81 MSSA SAB vs 89 MSSA IE	MLST+DNA microarray 185 genes	None	No association between CC or virulence genes and IE. Intravenous drug use associated to CC8.
Rasmussen *et al.* [6]	46 *S. aureus* nasal carriers vs 55 SAB vs 33 IE	MLST+DNA microarray 170 genes	None	CC30 associated to nasal carriage. Invasive *S. aureus* associated to 4 genes: *cap5, sasG, fnbB, LukD/LukE.*
Nethercott *et al.* [8]	24 *S. aureus* STI* vs 49 SAB vs 24 IE	MLST+PCR microarray 185 genes	None	PERMANOVA showed CC12+CC20 associated to IE. IE associated to 8 genes: *ssl03, ss17-set1, ssl8-set12, ssl08, ssl9-set5, setB3, fib, lukY-var1.*
Bouchiat *et al.* [11]	54 CA-SAB vs 72 native valve CA-IE	Multiplex PCR+microarray+DAPC	Bonferroni	CC15 associated to IE. Univariate genetic markers with no association to IE. Combination of 8 specific genetic markers showed subtle association to IE. No phenotypic differences.

*STI, Skin and soft tissue infection.

Our study has some of the same limitations as the previous studies. The primary concern is statistical power. The first question is how large a difference is needed to infer clinical importance? None of the previous studies have addressed this problem by performing power calculations. The size of our population is comparable to the largest of the previous studies, with none of them including more IE patients. Earlier studies identifying statistical significant differences in presence/absence of virulence genes have found proportion differences around 30 %. Our power calculation showed that we would be able to detect significant SNP proportion differences of the same size. It is likely that doubling our sample size would have made several smaller differences statistically significant. This raises the issue of how relevant it is to identify subtle genetic differences, e.g. a SNP being present in 40 % of SAB-only isolates vs 60 % of IE isolates.

The next concern is that different clinical patient groups are included in the existing studies making direct comparisons difficult. The study most similar to our present one with regard to patient inclusion, did not find any genetic associations to IE either [10]. In order to make the population more homogenous we carried out a subgroup analysis of CA native valve disease. Despite the fact that the subgroup had approximately the same size of a similar study [11], reduced power could be the reason why we did not identify any significant differences.

Another important issue is multiple testing. If many genes are investigated, significant differences may occur just by chance if the results are not corrected for multiple testing. The studies investigating up to 185 genes without correcting for multiple testing are prone to find around nine genes to differ significantly just by chance. A strength of our study is the thorough correction for multiple testing with both FDR and/or Bonferroni.

While the earlier studies of clinical association have investigated only genes covered by the specific microarray assay, a strength of our setup is WGS currently being the least biased approach to investigate genetic associations. However, there are limitations to this approach as well. SNP analyses are dependent on a reference genome and no single reference will allow the capture of all differences present since the dataset consists of multiple different CCs. This could be overcome by focusing on a single CC, but that would limit the generalisability of the findings. The present study adjusts for lineage effects by performing regression along the CCs and analysing the residuals. While this approach only corrects for lineage effects giving rise to the different CCs and not for lineage effects within the groups it has proven successful when analysing associations in other data sets. We used it effectively to reduce the signal from lineage effects and identify individual genes and SNPs giving rise to antimicrobial resistance in a previously published data set of *Escherichia coli* also analysed by [19] (data not shown).

As in the earlier studies a relevant limitation is that we only characterised a single isolate from the bloodstream of each SAB-only and IE patient. In this way, there may be a subpopulation of IE prone variants that are overlooked. A way to address this would be to sequence a number of bloodstream isolates and/or heart valve isolates from patients undergoing cardiac surgery.

Last but not least is the question of how we should interpret the current controversy in the literature? A possible explanation is that the results are influenced by different study designs with heterogeneous patient populations and geographical variation. Despite this, it is vital that results are reproducible to a certain extent in order to provide evidence that there are clear genetic differences between isolates causing IE and isolates causing SAB-only.

### Conclusion

Based on our study and the current literature, it is not convincing that a specific *S. aureus* genotype is associated to IE in patients with *S. aureus* bacteraemia.

With extensive genetic analyses using WGS, we found no genetic association to IE suggesting that any *S. aureus* isolate as a true opportunistic pathogen may have the capacity to undergo transition from a bloodstream infection to an endocardial infection given the right circumstances in a given host. Weaker bacterial genetic signals or signals overshadowed by the clonal nature of *S. aureus* may still be involved, but this will need larger clone-specific studies to determine.

## Data bibliography

1. The Wellcome Trust Sanger Institute. European Nucleotide Archive ERP023934 (2017).
2. Lilje B, Johannesen TB. Sourceforge https://sourceforge.net/projects/sabvsie/files/ (2017).
3. Stegger M, Driebe EM, Roe C, Lemmer D, Bowers JR *et al.* GenBank NC_021554.1 (2013).

## Supplementary Data

1649 Supplementary File 1
