# Supplementary material for: Whole-genome sequencing of bloodstream Staphylococcus aureus isolates does not distinguish bacteraemia from endocarditis
Source: Microb Genom. 2017 Nov 20;3(11):e000138. doi: 10.1099/mgen.0.000138 (PMC5729915; doi:10.1099/mgen.0.000138)
Supplement: Supplementary File 1 [file mgen-3-138-s001.pdf]

Supplementary material

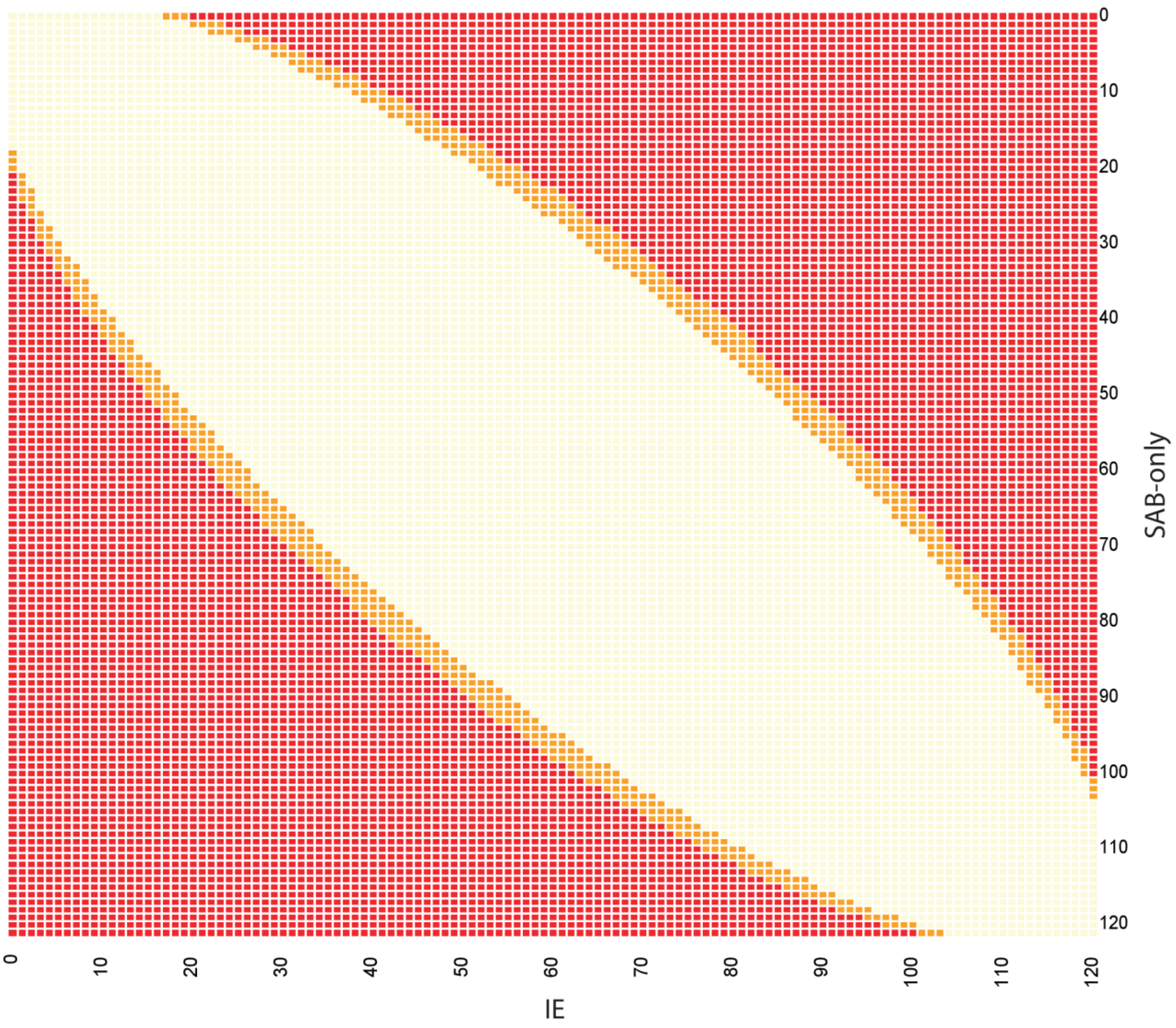

Figure S1

Powerplot showing: the number of SAB-only and IE isolates that have to contain a given SNP for a Fisher test to be significant after multiple testing correction (Bonferroni).

Each square corresponds to a SNP being present in X number of IE- and Y number of SAB-only isolates. E.g. the lower left square indicates a SNP being present in one IE isolate and 241 SAB-only isolates. The color of the square corresponds to significance level. Red squares correspond to a p-value <0.05 after multiple testing correction, orange squares correspond to p-values >0.05 and < 1 after correction, light yellow squares correspond to p-values =1 after multiple testing correction.

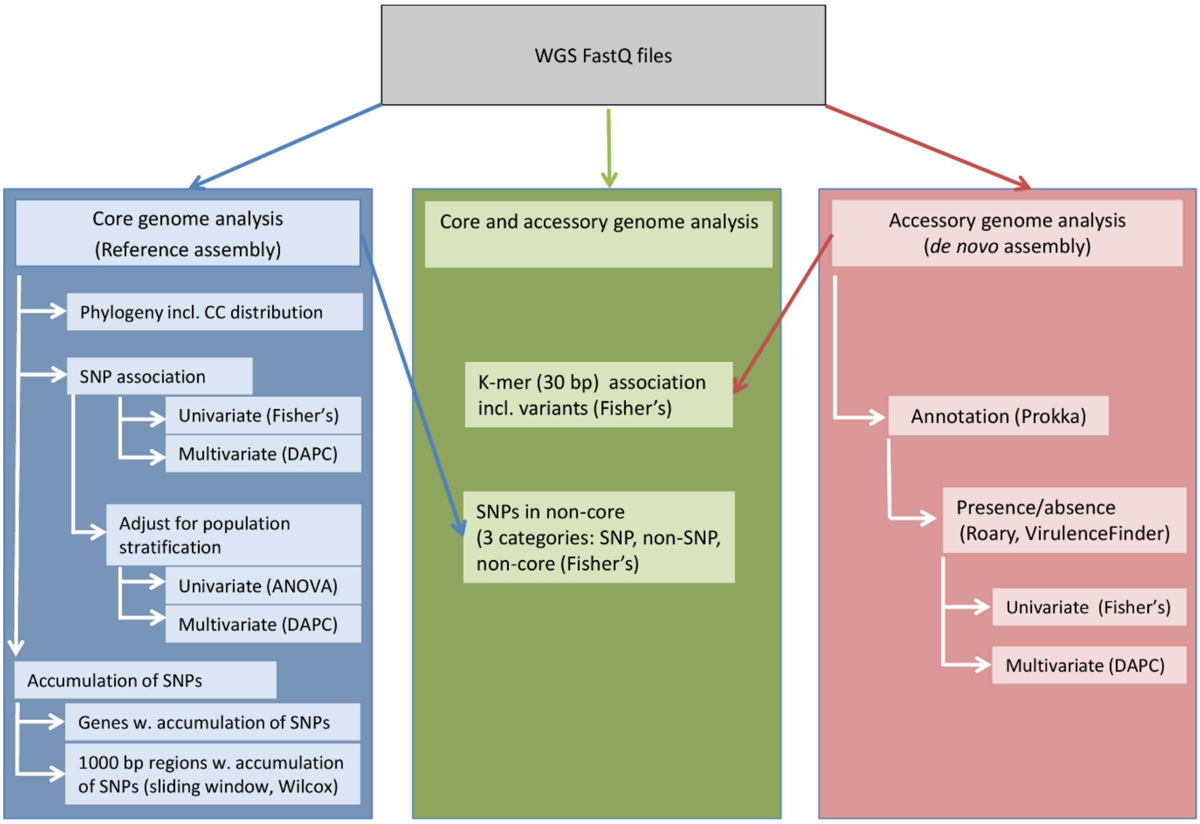

Figure S2

Flow chart of the bioinformatics methods applied in this study.

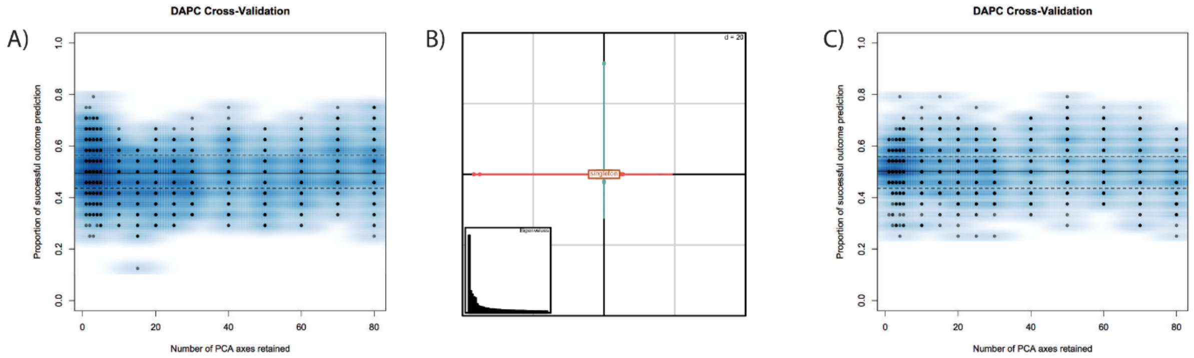

Figure S3

Multivariate analysis: A) Cross validation from DAPC, B) PCA after population stratification, samples colored by CC type (comparable to fig 3. A), due to lineage correction each CC will average around the same spot. Eigenvalues are shown in the plot; five axes were kept for PCA analysis, the first two values are plotted C) Cross validation analysis after population stratification.

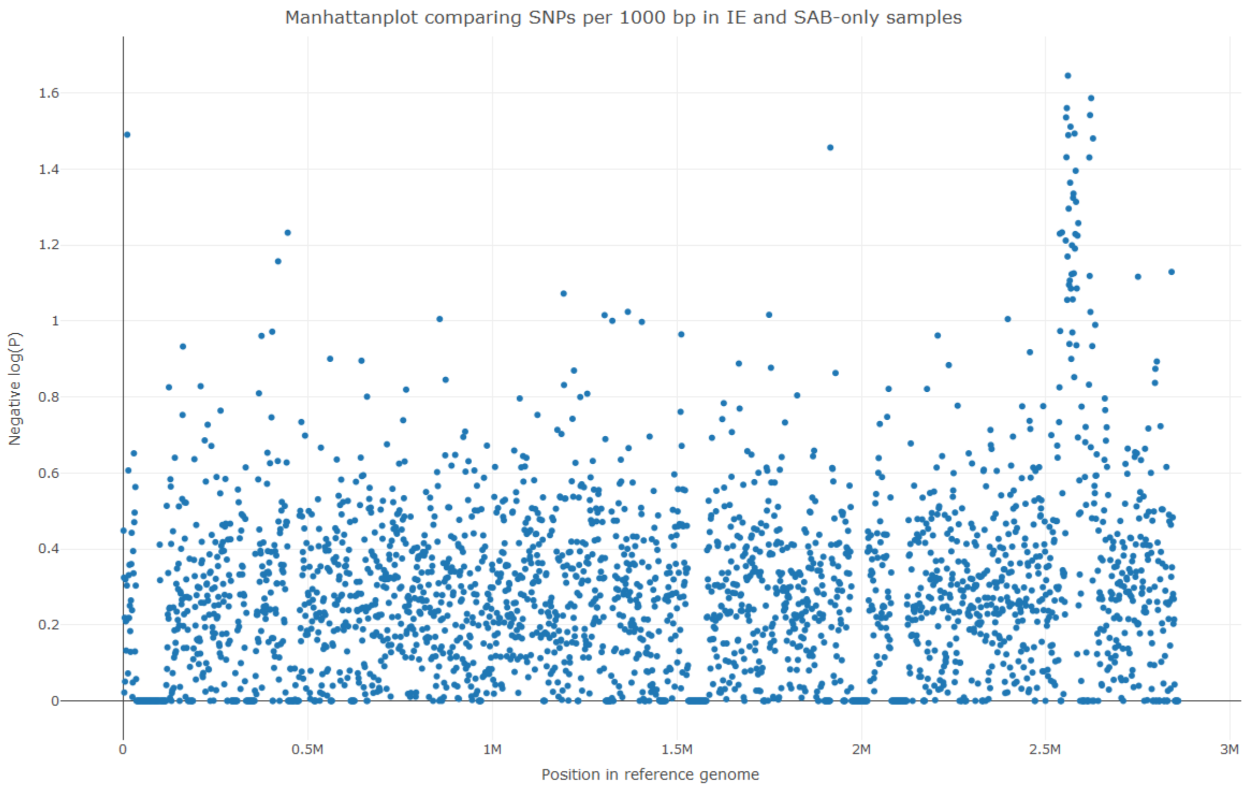

Figure S4

Manhattan plot of the full reference genome. The reference genome is split into 1000 bp bins and P-values are based on a t-test comparing the number of SNPs in each bin between SAB and IE samples.

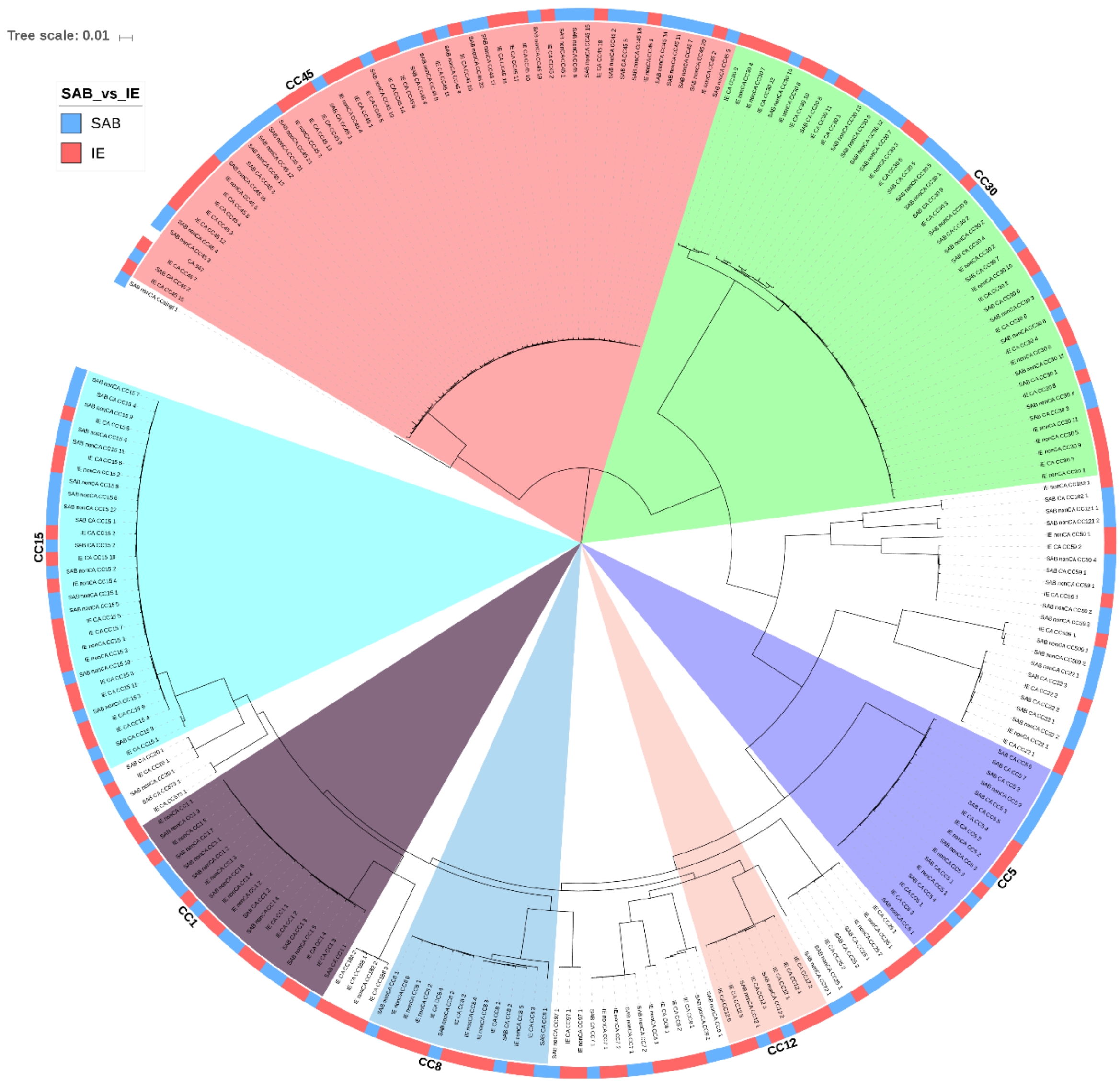

Figure S5

Rooted phylogenetic analysis using a maximum likelihood approximation on the 120.850 SNPs identified in the conserved core genome of the 241 S. aureus isolates. The tree is rooted on the CC45 branch (Driebe et al. 2015), and CC groups with more than 10 isolates are highlighted. Outer circle corresponds to infection type (red=IE, blue=SAB-only)

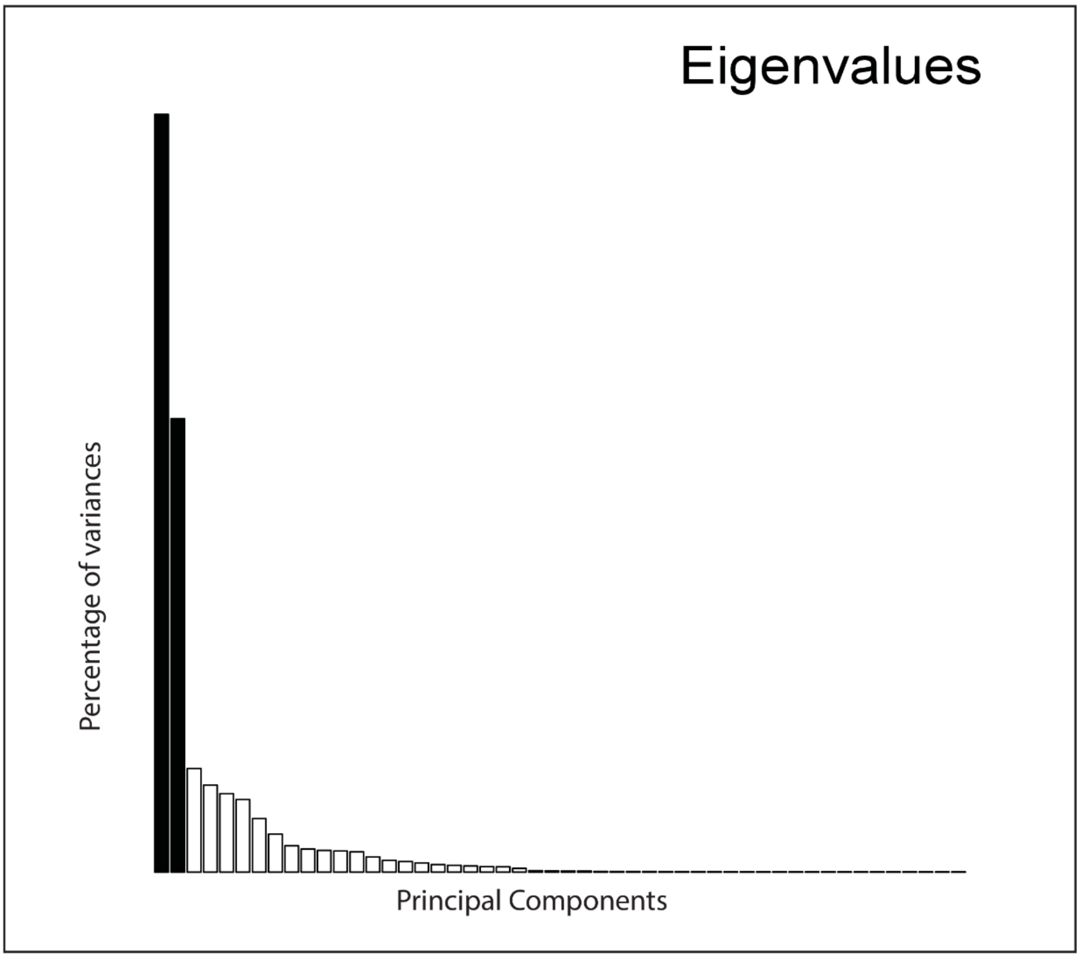

Figure S6

Bar plot of the Eigenvalues obtained from the PCA analysis in Fig. 3. The X-axis shows the principan components, the Y-axis the percentage of variance accounted for by each PC. Only the two first axis (in black) were kept for PCA analysis and plotted.
